# Temporal trends, risk factors and outcomes of infections due to extended-spectrum β-lactamase producing *Enterobacterales* in Swiss solid organ transplant recipients between 2012 and 2018

**DOI:** 10.1186/s13756-021-00918-7

**Published:** 2021-03-07

**Authors:** Philipp Kohler, Aline Wolfensberger, Susanne Stampf, Andreas Brönnimann, Katia Boggian, Christian van Delden, Melody Favre, Cédric Hirzel, Nina Khanna, Stefan P. Kuster, Oriol Manuel, Dionysios Neofytos, Silvio Ragozzino, Peter W. Schreiber, Laura Walti, Nicolas J. Mueller, Swiss Transplant Cohort Study, Patrizia Amico, Andres Axel, John-David Aubert, Vanessa Banz, Beckmann Sonja, Guido Beldi, Christian Benden, Christoph Berger, Isabelle Binet, Pierre-Yves Bochud, Sanda Branca, Heiner Bucher, Thierry Carrel, Emmanuelle Catana, Yves Chalandon, Sabina de Geest, Olivier de Rougemont, Michael Dickenmann, Joëlle Lynn Dreifuss, Michel Duchosal, Thomas Fehr, Sylvie Ferrari-Lacraz, Christian Garzoni, Paola Gasche Soccal, Christophe Gaudet, Emiliano Giostra, Déla Golshayan, Karine Hadaya, Jörg Halter, Dimitri Hauri, Dominik Heim, Christoph Hess, Sven Hillinger, Hans Hirsch, Patricia Hirt, Günther Hofbauer, Uyen Huynh-Do, Franz Immer, Michael Koller, Bettina Laesser, Brian Lang, Roger Lehmann, Alexander Leichtle, Christian Lovis, Oriol Manuel, Hans-Peter Marti, Pierre Yves Martin, Michele Martinelli, Katell Mellac, Aurélia Merçay, Karin Mettler, Pascal Meylan, Nicolas Mueller, Antonia Müller, Thomas Müller, Ulrike Müller-Arndt, Beat Müllhaupt, Mirjam Nägeli, Manuel Pascual, Klara Posfay-Barbe, Juliane Rick, Anne Rosselet, Simona Rossi, Silvia Rothlin, Frank Ruschitzka, Urs Schanz, Stefan Schaub, Aurelia Schnyder, Macé Schuurmans, Federico Simonetta, Katharina Staufer, Susanne Stampf, Jürg Steiger, Guido Stirniman, Christian Toso, Christian Van Delden, Jean-Pierre Venetz, Jean Villard, Madeleine Wick, Markus Wilhelm, Patrick Yerly

**Affiliations:** 1 Division of Infectious Diseases and Hospital Epidemiology, Cantonal Hospital St. Gallen, St. Gallen, Switzerland; 2 Division of Infectious Diseases and Hospital Epidemiology, University Hospital Zurich and University of Zurich, Zurich, Switzerland; 3 Clinic for Transplantation Immunology and Nephrology (Swiss Transplant Cohort Study), University Hospital of Basel, Basel, Switzerland; 4 Transplant Infectious Diseases Unit, Faculty of Medicine, University Hospitals Geneva, Geneva, Switzerland; 5 Department of Infectious Diseases, Bern University Hospital, University of Bern, Bern, Switzerland; 6 Division of Infectious Diseases and Hospital Epidemiology, University and University Hospital Basel, Basel, Switzerland; 7 Infectious Diseases Service and Transplantation Center, Lausanne University Hospital (CHUV), Lausanne, Switzerland

**Keywords:** Solid organ transplant, Renal transplant, Extended-spectrum beta-lactamase, Enterobacterales, Switzerland

## Abstract

**Background:**

The burden of antimicrobial resistance is high in solid organ transplant (SOT) recipients. Among Swiss SOT recipients, we assessed temporal trends of ESBL-producing *Enterobacterales* (ESBL-E), identified risk factors for ESBL-E, and assessed the impact of resistance on patient outcome.

**Methods:**

Data from the Swiss Transplant Cohort Study (STCS), a nationwide prospective cohort of SOT-recipients, were analysed. Temporal trends were described for ESBL-detection among *Escherichia coli* and non-*Escherichia coli*. In a nested case–control study, cases with ESBL-E infection were 1:1 matched (by time since transplantation, organ transplant, pathogen) to controls infected with non-ESBL-E. Factors associated with resistance and with unfavourable 30-day outcome (death, infection relapse, graft loss) were assessed.

**Results:**

From 2012 to 2018, we identified 1′212 infection episodes caused by *Enterobacterales* in 1′074 patients, thereof 11.4% (138/1′212) caused by ESBL-E. The proportion of ESBL-production among *Escherichia coli* remained stable over time (*p* = 0.93) but increased for non-*E. coli* (*p* = 0.02) *Enterobacterales*. In the case–control study (n = 102), antibiotic pre-treatment was independently associated with ESBL-production (aOR = 2.6, 95%-CI: 1.0–6.8, *p* = 0.046). Unfavourable outcome occurred in 24/51 (47%) cases and 9/51 (18%) controls (*p* = 0.003). Appropriate empiric antibiotic therapy was the only modifiable factor associated with unfavourable outcome.

**Conclusions:**

In Swiss SOT-recipients, proportion of infections with ESBL-producing non-*E. coli Enterobacterales* increased in recent years. Antibiotic pre-treatment represents a risk factor for ESBL-E. Improving appropriateness of empiric antibiotic treatment might be an important measure to reduce unfavourable outcome, which was observed in almost half of SOT-recipients with ESBL-E infections.

## Introduction

Antimicrobial resistance is a major threat to the achievements of modern medicine. Solid organ transplant (SOT) recipients are at particular risk for acquisition of resistant pathogens, most likely due to increased healthcare and antibiotic exposure [1]. Specifically in SOT-recipients, donor-derived infections due to resistant pathogens are associated with significant burden of disease [2]. Over the last decade, Gram-negative bacteria have become the focus of attention regarding antibiotic resistance for both the general hospital population and SOT-recipients. A recent systematic review reported that 20% of SOT-recipients are colonized with ESBL-producing *Escherichia coli* [3]. Colonization by ESBL-producing isolates is an important risk factor for subsequent infection [4]; about one in 10 renal transplant recipients (RTR) colonized by ESBL-producing bacteria experiences a urinary tract infection (UTI) caused by these pathogens [5]. Compared to infections with non-resistant pathogens, those caused by resistant bacteria are associated with an increased risk for recurrent infection, allograft dysfunction and excess mortality [5, 6]. Case-fatality infection with resistant pathogens is high (up to 50% in the case of bacteremia due to carbapenem-resistant *Enterobacterales*) [7–9].

Among Swiss SOT recipients, most infections in the first year post-transplantation are caused by *Enterobacterales.* Among *E. coli* and *Klebsiella pneumoniae*, ESBL-production was observed in 15%, whereas no carbapenemase-producing *Enterobacterales* (CPE) were identified in our cohort [10]. Here, we aimed to assess temporal trends of ESBL-producing *Enterobacterales* (ESBL-E), to identify risk factors for infections with ESBL-E, and to assess the impact of ESBL-production on patient outcome.

## Methods

### Data source

The Swiss Transplant Cohort Study (STCS) is a nationwide, multi-centre, open, prospective cohort and has enrolled all SOT-recipients in Switzerland since May 2008 [11]. Clinical and laboratory data are prospectively collected at the time of transplantation, at 6 and 12 months, and annually thereafter. Infectious episodes are identified by transplant infectious disease (ID) physicians on a regular basis using electronic patient records, according to definitions developed by the STCS infectious diseases working group [10]. Six participating transplantation centres (Basel, Bern, Geneva, Lausanne, St. Gallen and Zurich) undergo regular data monitoring and in-depth data quality audits.

### Study design and participants

We included SOT-recipients of heart, liver, kidney, and kidney-pancreas grafts aged 18 or older enrolled in the STCS. Lung transplant recipients were excluded because of the particular challenge to distinguish colonisation from infection in this population. From August 2012 onwards, information about ESBL production has been available in the STCS database. For the analysis of temporal trends, we thus retrieved all infection episodes diagnosed between August 2012 and December 2018. Participants contributed a maximum of one episode per year. Those with episodes caused by resistant and susceptible pathogens were counted as having had one resistant episode and were not eligible as controls. Patients with episodes caused by ESBL-producing *E. coli* and non-*E. coli Enterobacterales* in the same year were counted in both groups.

A nested case–control study was performed to assess risk factors for infection by ESBL-E and its effect on 90-day outcome afterwards. As opposed to the analysis for temporal trends (episode level) this analysis was performed on the patient-level (i.e. only one episode per patient considered). We included all patients with infections due to ESBL-producing (or MDR and extended-spectrum cephalosporine-resistant, see below) *Enterobacterales*, diagnosed between August 2012 and December 2016. In case of multiple episodes caused by a resistant pathogen, only the patients’ first episode after transplantation was considered. Cases were matched to controls in a 1:1 fashion, applying incidence density sampling according to time to first infectious episode after transplantation; type of transplanted organ and bacterial pathogen were used as further matching criteria. Controls with previous colonization by ESBL-E were excluded. Detailed information about infections in cases and controls were additionally collected via chart review and recorded in an electronic database (SecuTrial®). These included administration of antibiotics within 30 days before infection (both therapeutic and prophylactic), travel history, urinary obstruction (only for RTR), admission to acute or intensive care, involvement of ID specialist, type, duration and effectiveness of empiric and definite antibiotic treatment, and 90-day outcome.

### Microbiology

Pathogen identification and resistance testing was performed on a routine basis in the microbiology laboratories serving the participating centres. Since 2012, information about infections caused by ESBL-E is being recorded. Also, the presence of multidrug-resistance (MDR) is recorded according to the European Centre for Disease Prevention and Control (ECDC) definitions [12]. 

For the analysis of temporal trends, the variable “ESBL-production” as reported in the database was used to classify resistant and susceptible pathogens. For the case–control study, this definition was extended to also include MDR pathogens with resistance to extended-spectrum cephalosporins (ESC), i.e. 3rd or 4th generation cephalosporins. This approach was chosen because for some bacterial isolates ESBL-production was not tested or reported.

### Definitions

In brief, proven infection was defined as the presence of clinical signs or symptoms, detection of a bacterial pathogen, and given treatment [10]. Effectiveness of antibiotic treatment was assessed according to locally performed susceptibility tests. Beta-lactam/beta-lactamase inhibitor combinations were considered inadequate against ESBL-E irrespective of the reported minimal inhibitory concentration. Unfavourable outcome was defined as any of the following: microbiological relapse (i.e. infection with the same pathogen at same body site as the initial infectious episode), graft failure (defined as dialysis post renal transplantation; or re-transplantation post heart or liver transplantation; or recurrence of insulin-dependence following pancreas transplant) or death, all within 90 days after infection.

### Statistical analysis

A temporal trend analysis was performed to detect a pattern in occurrence of infectious episodes caused by ESBL-producing *E. coli* and non-*E. coli Enterobacterales* between 2012 and 2018, using Chi-squared test for trends in proportion. In the matched subpopulation (including episodes which occurred between 2012 and 2016), a descriptive analysis was done followed by univariate and multivariable conditional logistic regression to evaluate risk factors for infections caused by ESBL-E. Age and gender, as well as baseline characteristics associated with ESBL-E infection in univariate analysis (*p* < 0.1) were included in the multivariable model. Multicollinearity was assessed calculating the variance inflation factor (cut-off > 10).

For the analysis regarding impact of resistance on patient outcome, infection by ESBL-E itself was considered a key predictor. We used the change in estimate method as screening method for selection of co-variables into the multivariable logistic regression model (i.e. change of key predictor estimate > 10% after adding the co-variable to the model) [13]. R software version 3.6.1 was used for all statistical analyses; a *p* value of < 0.05 was considered statistically significant.

## Results

### Temporal trends

**Fig. 1:**
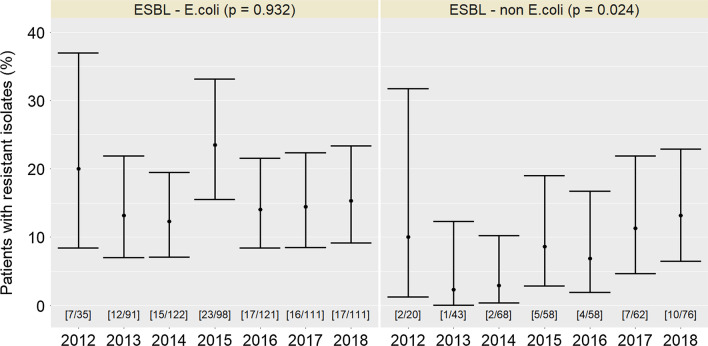
Proportion of patients with ESBL-producing *Escherichia coli* (left) and non-*E. coli* (right) among all patients infected with the corresponding pathogen in the Swiss Transplant Cohort Study between 2012 and 2018

Between 2012 and 2018, we registered 1′212 infectious episodes caused by *Enterobacterales* among 1′074 patients, mostly among RTR (784/1′074, 73%). Among all isolates, ESBL-production was reported in 138/1′212 isolates (11.4%). ESBL rates for episodes of kidney, heart, and liver transplant patients were 13.2% (168/1271), 12.4% (11/89), and 13.6% (30/221), respectively; across the six participating centres, the proportion of ESBL-producing isolates ranged from 8.3 to 18.3%. The proportion of ESBL *E. coli* remained stable over time (*p* = 0.93) whereas an increasing trend (*p* = 0.024) was observed for non-*E. coli Enterobacterales* (Fig. 1). No temporal trend was observed when ESBL *E. coli* and non-*E. coli* were combined (*p* = 0.36).

### Case–control study: population and infection characteristics

Between 2012 and 2016, we identified 88 case patients which were matched to 88 controls. After chart review and revision of the original susceptibility test results, 51 matching pairs remained for the analysis. For the 37 excluded patients, reasons for exclusion were mostly limited or missing susceptibility testing (15/37) and revision of case status (14/37). Of the 51 cases and controls, 33 (65%) were RTR, 4 (8%) kidney-pancreas, 9 (18%) liver or kidney-liver, and 5 (10%) heart transplant patients, respectively. The urinary tract was the most common site of infection (75%). *E. coli* was the most frequent pathogen found in infections (75.5%), followed by *K. pneumoniae* (15.7%) and other pathogens (8.8%).

**Fig. 2:**
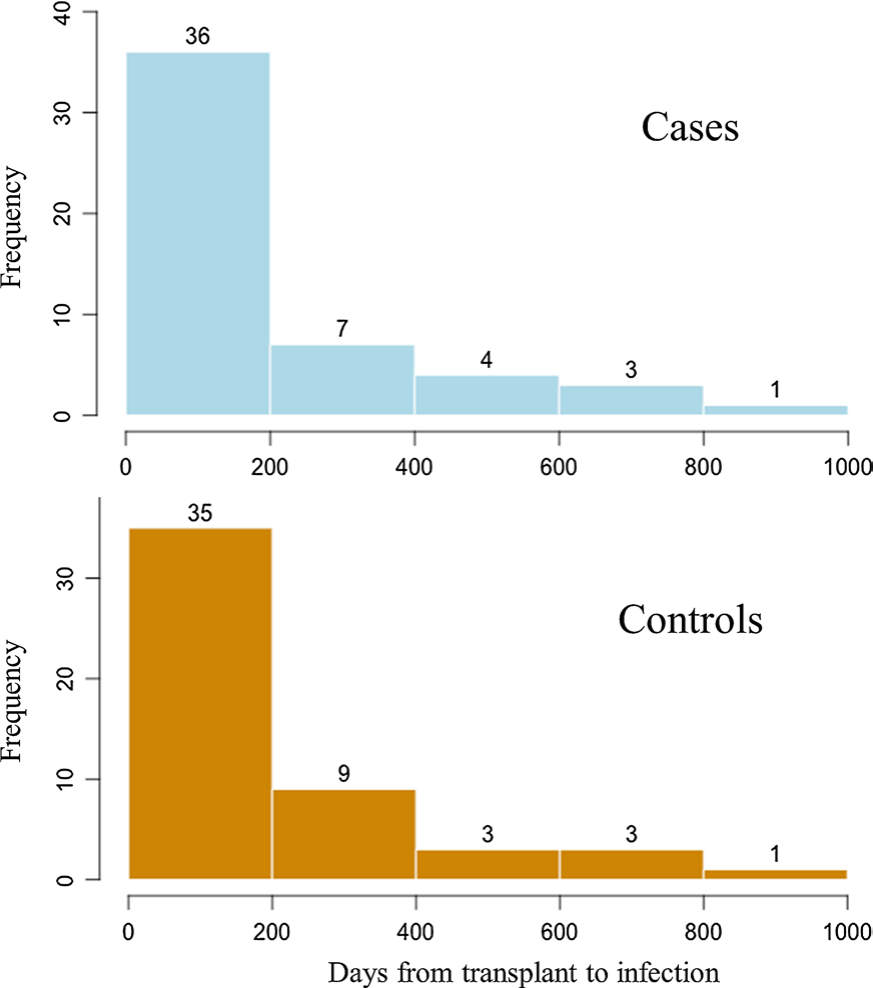
Histogram showing the time from transplant to onset of infection in days for 51 cases (above) and 51 controls (below)

Baseline characteristics of cases and controls were summarized in Table 1. Infections occurred a median of 69 days after transplant (interquartile range [IQR]: 25–232), mostly (68%) within the first 6 months after transplantation (Fig. 2). Patients were predominantly outpatients at time of diagnosis (63% for ESBL-E, 61% for non-ESBL-E infections). ESBL-E and non-ESBL-E infections were evenly distributed among centres and years, as were infection sites and comorbidities. Travel history was not available for most patients and therefore dropped from further analyses.

**Table 1:** Baseline characteristics of patients infected without and with extended-spectrum beta-lactamase (ESBL)-*Enterobacterales*

	Cases (N = 51)	Controls (N = 51)
Male, N (%)	28 (54.9%)	17 (33.3%)
Age at time of infection, mean (SD)	54.8 (12)	53.4 (14.8)
Transplanted organ (matched variable)		
Kidney	33 (64.7%)	33 (64.7%)
Kidney-pancreas	4 (7.8%)	4 (7.8%)
Liver (incl kidney-liver)	9 (17.6%)	9 (17.6%)
Heart	5 (9.8%)	5 (9.8%)
BMI at transplantation, mean (SD)	25.5 (4.1)	25.1 (4.2)
Caucasian ethnicity, N (%)	46 (90.2%)	49 (96.1%)
Comorbidities, N (%)		
Cardiopulmonary disease	27 (52.9%)	25 (49%)
Metabolic/endocrine disease	35 (68.6%)	43 (84.3%)
Cancer	8 (15.7%)	6 (11.8%)
Other	32 (62.7%)	31 (60.8%)
Previously documented ESBL colonization^a^	19 (37%)	0 (0%)
Antibiotics^b^ (30 days before infection), N (%)	24 (47.1%)	14 (27.5%)
BL/BLI	10 (37%)	6 (28.6%)
Carbapenem	6 (22.2%)	3 (14.3%)
Quinolone	10 (37%)	3 (14.3%)
Cephalosporine	7 (25.9%)	0 (0%)
Sulfamethoxazol/trimethoprim	7 (25.9%)	4 (19%)
Hospital-onset, N (%)	20 (39.2%)	19 (37.3)
Day of onset (median, IQR)	20 (9.5–60)	8 (6–42)
Days from transplantation to infection, median (IQR)	67 (24–228)	71 (33–227)
Year of 1st infection, N (%)		
2012	6 (11.8%)	8 (15.7%)
2013	9 (17.6%)	13 (25.5%)
2014	14 (27.5%)	12 (23.5%)
2015	16 (31.4%)	10 (19.6%)
2016	6 (11.8%)	8 (15.7%)
Infection site, N (%)		
Urinary tract	38 (74.5%)	39 (76.5%)
With bacteremia	5 (13.2%)	10 (25.6%)
Abdominal/liver	2 (3.9%)	3 (5.9%)
Respiratory tract	3 (5.9%)	5 (9.8%)
Surgical site	3 (5.9%)	4 (7.8%)
Primary bacteremia	2 (3.9%)	1 (2.0%)
Other	3 (5.9%)	1 (2.0%)
If urinary tract infection, N (%)		
Obstruction	2/38 (5.3%)	5/39 (12.8%)
Catheter	12/38 (31.6%)	17/39 (43.6%)

*ESBL* extended-spectrum beta-lactamase, *SD* standard deviation, *BMI* body mass index, *IQR* interquartile range, *BL/BLI* beta-lactam/beta-lactamase-inhibitor

^a^Previously ESBL colonized participants were not eligible for the control group

^b^Including prophylaxis

### Risk factors for ESBL infection

Male gender (*p* = 0.04) and antibiotic treatment within 30 days prior to infection (*p* = 0.05) were significantly associated with ESBL-E infection (Table 2). Antibiotic pre-treatment remained as independent risk factor for ESBL-E infection in multivariable analysis after correction for age, gender and underlying metabolic/endocrinologic disease (adjusted OR = 2.6, 95% CI: 1.0–6.8, *p* = 0.046).

**Table 2:** Univariable and multivariable conditional logistic regression analysis regarding risk of infection with ESBL-producing pathogen (vs. non-ESBL)

	Univariate OR [95%-CI] (*p* value)	Multivariable aOR [95%-CI] (*p* value)
Male versus female	2.4 [1.0–5.4] (*0.04*)	2.3 [0.9–5.0] (0.08)
Age (years) at time of infection	1.0 [0.9–1.0] (0.58)	0.9 [0.9–1.0] (0.99)
BMI at transplantation	1.0 [0.9–1.1] (0.92)	
Caucasian versus others	0.4 [0.1–2.1] (0.27)	
Comorbidities (yes/no)		
Cardiopulmonary disease	1.1 [0.6–2.3] (0.72)	
Metabolic/endocrine disease	0.5 [0.2–1.1] (0.10)	0.4 [0.2–1.1] (0.09)
Cancer	1.4 [0.4–4.4] (0.57)	
Other	1.1 [0.5–2.7] (0.82)	
Year of infection		
2012	–	
2013	1.2 [0.3–4.7] (0.83)	
2014	1.6 [0.3–7.1] (0.57)	
2015	2.5 [0.6–11.1] (0.23)	
2016	0.9 [0.2–5.3] (0.94)	
Antibiotics (yes/no) before infection^a^	2.4 [1.0–5.9] (*0.048*)	2.6 [1.0–6.8] (*0.046*)

*ESBL* extended-spectrum beta-lactamase, *(a)OR* (adjusted) odds ratio, *CI* confidence interval, *BMI* body mass index

^a^Any therapeutic or prophylactic antibiotic treatment within 30 days prior to infection

### Infection management and outcome

Patients with susceptible and resistant pathogens were similar in terms of proportion of hospital admission (16/51 *vs.* 14/51), intensive care admission (9/51 *vs.* 8/51), or involvement of an ID physician for choice of treatment (22/51 *vs.* 27/51) (Table 3). Length of stay was longer for those with resistant (median 18 days, IQR 8.2–30 days) *vs.* those with susceptible pathogens (median 9, IQR 3–24 days), but did not reach statistical significance (*p* = 0.10). Patients with susceptible pathogens were more likely to receive appropriate empiric antibiotic therapy (36/51, 76%) *vs.* those with resistant pathogens (16/51, 36%) (*p* < 0.001). Inappropriate therapy was mostly due to late initiation (i.e. > 2 days after infection) of antibiotic therapy (similar in both groups: 8/51 *vs.* 11/51, *p* = 0.44), or due to administration of beta-lactam/beta-lactamase inhibitors against resistant pathogens (1/51 *vs.* 10/51, *p* = 0.004).

**Table 3:** Management and outcome of infections caused by non-extended-spectrum beta-lactamase (ESBL)- and ESBL-producing *Enterobacterales*

	Cases (N = 51)	Controls (N = 51)	*p* value^a^
*Management*			
Hospital admission if community onset, N (%)	14 (43.8%)	16 (50.0%)	0.99
Length of stay after infection (median, IQR)	18 (8.2–30)	9 (3–24)	0.10
On intensive care after infection onset, N (%)	8 (23.5%)	9 (25.7%)	1.00
Days on intensive care (median, IQR)	5.5 (4–16)	3 (2–11)	0.38
Infectious diseases consult, N (%)	27 (52.9%)	22 (43.1%)	0.43
Initially	21 (41.2%)	16 (31.4%)	0.41
Upon microbiology results	18 (35.3%)	19 (37.3%)	1.00
Antibiotics (30 days after infection), N (%)			
Adequate empiric therapy^b^	16 (35.6%)	34 (75.6%)	< *0.001*
Inadequate BL/BLI^c^	10 (22.7%)	1 (2.3%)	*0.004*
Inadequate ceftriaxone/ceftazidime	2 (4.5%)	0 (0%)	0.15
Inadequate ciprofloxacin	5 (11.4%)	1 (2.3%)	0.09
No therapy before day 2	11 (21.5%)	8 (18.2%)	0.44
*Outcome*			
Re-admission, N (%)	15 (29.4%)	14 (27.5%)	1.00
Due to infection	12 (23.5%)	8 (15.7%)	0.35
Unfavourable outcome within 90 days, N (%)	24 (47.1%)	9 (17.6%)	*0.003*
Relapse	20 (39.2%)	5 (9.8%)	*0.001*
Graft loss	1 (2.0%)	4 (7.8%)	0.36
Death	4 (7.8%)	3 (5.9%)	1.00

*ESBL* extended-spectrum beta-lactamase, *IQR* interquartile range, *BL/BLI* beta-lactam/beta-lactamase-inhibitor

^a^Chi-square or Fisher’s exact test for dichotomous variables, as appropriate; Mann–Whitney U for continuous variables

^b^Administered within two days after infection diagnosis

^c^Inadequate BL/BLI were all piperacillin/tazobactam except for one case who received amoxicillin/clavulanic acid

Unfavourable outcome occurred in 9/51 (18%) controls with non-ESBL-E infections as compared to 24/51 (47%) patients with ESBL-E infections (*p* < 0.003). This difference was mainly due to relapse of infection (5/51 *vs.* 20/51, *p* = 0.001), whereas graft loss (4/51 *vs*. 1/51) and mortality (3/51 *vs.* 4/51) were similar in both groups.

Evaluating the impact of resistance on unfavourable outcome, infection with ESBL-E was associated with unfavourable (OR = 4.0, 95%-CI: 1.7–10.5, *p* = 0.003) and adequate empiric therapy (OR = 0.3, 95%-CI: 0.1–0.9, *p* = 0.03) with favourable outcome in univariate analysis (Table 4). After adjusting for modifiable (adequate empiric therapy) and non-modifiable (gender and need for ICU stay after infection) factors, the effect of ESBL-E infection was still large, but not anymore significantly associated with unfavourable outcome (adjusted OR = 3.1, 95%-CI: 0.8–12.5, *p* = 0.10).

**Table 4:** Risk of unfavourable outcome after *Enterobacterales* infection in solid organ transplant recipients

	Favourable (N = 69)	Unfavourable (N = 33)	Univariate		Multivariable	
			OR [95%-CI] (*p* value)		aOR [95%-CI] (*p* value)	
ESBL infection, N (%)	27 (37.9)	24 (74.3)	4.1 [1.7–10.7] (< *0.01*)		3.1 [0.8–12.5] (0.10)	
Age, mean (SD)	54.2 (13.7)	53.8 (13.1)	1.0 [0.9–1.0] (0.89)			
Male^**a**^, N (%)	32 (46.4)	13 (39.4)	0.8 [0.3–1.7] (0.51)		0.5 [0.1–1.5] (0.20)	
BMI at transplant (mean, SD)	25.0 (3.8)	25.9 (4.8)	1.0 [0.9–1.2] (0.33)			
Ethnicity: caucasian	66 (95.7)	29 (87.9)	0.3 [0.1–1.6] (0.16)			
Year of infection, N (%)						
2012	8 (11.6)	6 (18.2)	–			
2013	17 (24.6)	7 (21.2)	0.6 [0.1–2.2] (0.39)			
2014	18 (26.1)	6 (18.2)	0.4 [0.1–1.8] (0.26)			
2015	17 (24.6)	9 (27.3)	0.7 [0.2–2.7] (0.61)			
2016	9 (13.0)	5 (15.2)	0.7 [0.2–3.4] (0.70)			
Transplanted organ, N (%)						
Kidney	41 (59.4)	25 (75.8)	–			
Non-Kidney	28 (40.6)	8 (24.2)	0.4 [0.2–1.1] (0.09)			
Comorbidities, N (%)						
Cardiopulmonary disease	9 (13.0)	5 (15.2)	1.2 [0.5–2.9] (0.62)			
Metabolic/endocrine disease	34 (49.3)	18 (54.5)	1.2 [0.5–3.5] (0.70)			
Cancer	52 (75.4)	26 (78.8)	1.2 [0.4–3.8] (0.77)			
Other	43 (62.3)	20 (60.6)	0.9 [0.4–2.2] (0.87)			
Causing pathogen, N (%)						
*Escherichia coli*	54 (78.3)	23 (69.7)	–			
*Klebsiella* spp.	7 (10.1)	9 (27.3)	3.0 [1.0–9.4] (*0.05*)			
Other	8 (11.6)	1 (3.0)	0.3 [0.1–1.7] (0.26)			
Time to infection, median (IQR)	70 (31–256)	61 (14–203)	1.0 [1.0–1.0] (0.24)			
Type of infection, N (%)						
Urinary tract (vs. other)	50 (72.5)	27 (81.8)	1.7 [0.6–5.2] (0.31)			
Bacteremia	19 (27.5)	8 (24.2)	0.8 [0.3–2.1] (0.72)			
Hospital-onset, N (%)	26 (38.2)	13 (39.4)	1.1 [0.4–2.5] (0.91)			
LOS after infection^b^, median (IQR)	14 (4–26)	16 (7.2–33)	0.9 [0.9–1.0] (0.58)			
On ICU after infection^a^, N (%)	10 (21.3)	7 (31.8)	1.7 [0.5–5.4] (0.35)		2.3 [0.6–8.1] (0.20)	
Antibiotic therapy^c^, N (%)	60 (87.0)	28 (84.8)	0.8 [0.3–2.9] (0.77)			
Treatment with carbapenems	23 (33.3)	17 (51.5)	2.1 [0.9–5.0] (0.08)			
Treatment duration, median (IQR)	13 (7–19)	13 (7–18)	1.0 [0.9–1.0] (0.95)			
Adequate empiric therapy^a,d^, N (%)	39 (65.0)	11 (39.3)	0.3 [0.1–0.9] (*0.03*)		0.5 [0.1–1.7] (0.27)	
Infectious diseases consult, N (%)	36 (52.2)	13 (39.4)	0.6 [0.3–1.4] (0.23)			
Initially	27 (39.1)	10 (30.3)	0.7 [0.3–1.6] (0.39)			
Upon microbiology results	27 (39.1)	10 (30.3)	0.7 [0.3–1.6] (0.39)			

*BMI* body mass index, *ESBL* extended-spectrum beta-lactamase, *(a)OR* (adjusted) odds ratio, *CI* confidence interval, *IQR* interquartile range, *LOS* length of stay, *ICU* Intensive Care Unit

^a^Included in multivariable analysis based on individual impact on key predictor variable "ESBL infection"[13]

^b^Variable dropped from final model because of parsimony reasons; inclusion does not alter results

^c^Within 30 days after diagnosis

^d^Within 2 days after diagnosis

## Discussion

In this study based on data from a prospective national cohort representing all SOT-recipients in Switzerland, we show that ESBL-producing non-*E. coli* infections have been increasing over the last years and that antibiotic pre-treatment is independently associated with infection caused by ESBL-producing pathogens. Almost half of patients with ESBL-E had a relapsing infection compared to only 18% in those with non-ESBL E infections. Adequate empiric therapy, being less common among those with ESBL-E infection, was the only modifiable factor associated with unfavourable outcome. The comprehensive dataset and the thorough revision of the original data are particular strengths of the study.

The three last decades have witnessed a global dissemination of ESBL-producing *Enterobacterales* into healthcare systems and healthy populations alike [14]. From an epidemiological perspective, their emergence is propelled by various factors. Important drivers of community-acquisition (mostly ESBL-*E. coli* carrying the plasmid-encoded *bla*_CTX-M_ gene) are travel to/healthcare in endemic countries or household crowding [15, 16]. In contrast, ESBL-non *E. coli* (mostly *Klebsiella* spp.) are often hospital-acquired, mainly as a result of clonal expansion due to person-to-person transmission [17]. In our cohort of SOT-recipients, we found stable numbers or ESBL-*E. coli*, but an increase in ESBL-non *E. coli* over time, a worrisome finding which has also been observed in other European SOT cohorts [18–20]. We can only speculate as to the reason for this trend. Increased in-hospital transmission of these pathogens is one possible explanation, given the substantial differences in the prevalence of ESBL-producing *Enterobacterales* among participating centres in our study and given the many reports of resistant *K. pneumoniae* (and particularly high-risk clones such as ST11 or ST147) as a cause of nosocomial outbreaks among transplant and non-transplant patients [21–24].

In both community and hospital settings, antibiotic treatment—mainly cephalosporins but also quinolones—is associated with ESBL-colonization or infection, probably due to selection (or co-selection in the case of quinolones) of ESBL-E in the gut flora of colonized patients [15, 25]. Razazi et al*.* found treatment with 3rd generation cephalosporins to be predictive of ESBL-E colonization in patients admitted to intensive care [26]. In a Canadian cohort of RTR, antibiotic pre-treatment was the strongest risk factor for detection of resistant Gram-negative bacteria in the urine [27]; in a recent study on enterobacterial bloodstream infections among SOT-recipients in the United States, antibiotic exposure to trimethoprim-sulfamethoxazole and again 3rd generation cephalosporins were strong risk factors for infections with ESBL-producers [28]. We could confirm this important finding in our cohort of SOT-recipients. Due to the small sample size of the subgroups in our cohort, it is difficult to tell which antibiotic substances contributed mainly to this effect. It is important to note that not only cephalosporins or quinolones, but also beta-lactam/beta-lactamase inhibitor combinations have been independently associated with ESBL-*Klebsiella* spp., at least in hospitalized non-transplant patients [29]. Consequently, we think that antibiotic stewardship programs aiming at reducing the overall antibiotic use in this population is key in lowering the selection pressure for ESBL-E and other resistant pathogens. In this context it is important to note that—according to a recent survey among European transplant centres—a majority of transplant physicians uses antibiotics including quinolones and cephalosporins for asymptomatic bacteriuria in RTR despite the uncertain benefit of this intervention [30, 31].

In general, SOT-recipients infected with resistant pathogens fare worse than those infected with susceptible bacteria. Delmas-Frenette et al*.* found resistance to be associated with a longer duration of antibiotic treatment and a higher rate of hospitalization [27]. Other studies have even reported higher case fatality rates in those with resistant bacterial infections [6, 7]. In our univariable analysis, ESBL-production was strongly associated with unfavourable outcome, which was mostly recurrent infection among RTR. Similarly, data from a systematic review showed that UTI recurrence was clearly more common among RTR infected with ESBL-E compared to non-ESBL-E [5]. There are different reasons which could explain the higher recurrence rate in those with ESBL-E infections. First, as shown above, ESBL-infection develops more often in those with previous antibiotic treatment. This is probably the first step in a vicious circle, as antibiotic treatment itself might increase the risk for UTI recurrence which then again exposes the patient to antibiotic treatment [32]. Breaking this circle could be achieved by a reduction of antibiotic use. At least for the prevention of UTIs with non-antibiotic substances studies have shown promising results for transplant and non-transplant patients [33, 34]. Second, there have been suggestions that *E. coli* strains like ST131, a hyperendemic clone often associated with ESBL-production, come along with increased virulence compared to susceptible pathogens [35]. However, in a study among healthy young women ST131 was not associated with recurrence [36]. Third, patients with resistant infections are less likely to receive appropriate empiric antibiotic therapy, as shown in our results and by others [7]. In a recent study on community-acquired UTI among non-transplant patients, the higher recurrence rate among ESBL-E infections was primarily driven by inappropriate antibiotic treatment [37]. This is in line with results from our multivariable analysis, showing a reduction of the association between ESBL and unfavourable outcome after adjusting for inappropriate empiric therapy. Improving appropriateness of empiric therapy in this population without universally administering carbapenems represents a challenge. However, using clinical prediction tools which identify patients at high risk for ESBL-E infection could be an option [38]. Also, shortening the turnaround time of resistance results might mitigate the deleterious effect of inappropriate empiric therapy.

Limitations of our study are the retrospective design and the lack of travel history, which in the healthy population is among the most important risk factors for ESBL-E colonization and infection. However, travel is unlikely to have a major impact in this particular patient population. Because most of our study participants were RTR, the results might not be applicable to other SOT recipients. The sample size for the analysis of unfavourable outcome might have been too small to draw valid conclusions. In particular, our study might have been underpowered to evaluate the impact of being a RTR (compared to other SOT recipients), of infections with *K. pneumoniae* (vs. *E. coli*), or of treatment with carbapenems, which were all marginally associated with unfavourable outcome in univariate analysis. Also, molecular analysis of causing pathogens, which could shed light on the molecular epidemiology of ESBL-E in our geographic area including the presence of *E. coli* ST131 or healthcare-associated *K. pneumoniae* clones, was not performed. Further, using ESC-resistance as a proxy for ESBL-production is debatable. However, in the antibiotic resistance report of the ECDC from 2016, 89% of ESC-resistant *E. coli* were ESBL-producers [39]. Last, appropriateness of antibiotic therapy was defined in a rather conservative way, categorizing treatment with piperacillin/tazobactam as inappropriate also in non-bacteremic urinary tract infections.

## Conclusions

To conclude, ESBL-production among non-*E. coli Enterobacterales* has steadily been increasing among Swiss SOT-recipients in recent years. The role of resistant high-risk clones in this worrisome trend remains unknown. The only modifiable factor associated with the occurrence of ESBL-producing pathogens in our study was antibiotic pre-treatment, calling for action to strengthen antibiotic stewardship programs in this setting. Also, improving appropriateness of empiric antibiotic treatment might be an important measure to reduce unfavourable outcome, which occurred in almost half of SOT-recipients with ESBL-E infections.

## Data Availability

The datasets generated during and/or analysed during the current study are not publicly available but are available from the corresponding author on reasonable request.

## Abbreviations

CI: Confidence interval
CPE: Carbapenemase-producing *Enterobacterales*
ECDC: European Centre for Disease Prevention and Control
ESBL: Extended-spectrum beta-lactamase
ESBL-E: ESBL-producing *Enterobacterales*
ESC: Extended-spectrum cephalosporin
ICU: Intensive Care Unit
ID: Infectious diseases
IQR: Interquartile range
MDR: Multidrug-resistance
RTR: Renal transplant recipient
OR: Odds ratio
SOT: Solid organ transplant
STCS: Swiss Transplant Cohort Study
UTI: Urinary tract infection
